# Diffusion anomaly in nanopores as a rich field for theorists and a challenge for experimentalists

**DOI:** 10.1038/s41467-024-49821-w

**Published:** 2024-07-08

**Authors:** Stefano Brandani, Seungtaik Hwang, Jörg Kärger, Enzo Mangano

**Affiliations:** 1 School of Engineering, University of Edinburgh, Edinburgh, UK; 2 Faculty of Physics and Earth System Sciences, University of Leipzig, Leipzig, Germany

**Keywords:** Porous materials, Nanoscale materials

**arising from** J. Yuan et al. *Nature Communications* 10.1038/s41467-023-37455-3 (2023)

Diffusion in nanoporous materials directly affects matter upgrading by separation and catalytic conversion and in principle ultra-fast diffusion can enhance productivity and reduce costs. Yuan et al.^1^ reported the occurrence of ultra-fast diffusion in one-dimensional channels of a zeolite based on molecular simulations and uptake rate measurements. A careful re-analysis of the uptake data shows that experimental validation of ultra-fast diffusion by uptake rate measurements is yet to be confirmed. This implies that the potential enhancements in productivity and cost reduction cannot be demonstrated.

The quest to find conditions for enhanced diffusion in nanoporous materials is not new. Already half a century ago, Gorring was most likely the first to report the enhanced diffusion rates for chain molecules over a specific chain length^2^. On recording the rate of molecular uptake of the C_2_–C_14_ linear alkanes in a zeolite of structure type T, he noted that chain length enhancement from C_8_ till C_12_ leads to increasing rather than decreasing diffusivities.

Today, however, we know that these measurements (with large samples (5.3 g), small crystals ($< 1\,\mu m$) and large pressure steps (0–1 atm)) were made under conditions where the rate of sorption cannot be determined by intracrystalline diffusion^3,4^.

Nevertheless, Gorring’s paper stimulated numerous researchers to explore the conditions, under which guest molecules are subject to in nanoporous host systems, that may give rise to diffusivities rising rather than falling with increasing molecular sizes. This search was particularly fruitful in the field of molecular modeling where a couple of mechanisms leading to such anomalies could be identified. The terms chosen for their characterization are often nice nutshell descriptions of the underlying operational principles. Examples include commensurate adsorption^5^, incommensurate diffusion^6^, and resonant diffusion^7^, with each of them depending on the special fit of the guest molecules with the inner surface of the host material.

A very special effect of the host–guest interaction has been foreseen by Derouane et al.^8^ on considering diffusion in channel-like pores. Being subject to interaction with the surface of a host pore along the whole of its circumference, guest molecules of a proper size may be expected to move along the centerline of a pore, while smaller molecules cling to the pore walls. Once again, size enhancement may thus be found to give rise to diffusion enhancement. Originally termed as the floating molecule effect, today this phenomenon is generally referred to as the levitation effect and the possibility of its existence has been proven in numerous molecular dynamic simulations^9,10^.

As a result, there is indeed weighty evidence provided by molecular dynamics simulations that an enhancement of the size of the guest molecules in nanoporous materials may, under certain conditions, lead to enhanced rather than to diminished diffusivities.

In contrast, the yield achieved so far by experimental work in observing this diffusion anomaly is much less rewarding. This is mainly caused by the fact that in the experiment usually phenomena are investigated that are not solely determined by intracrystalline diffusion, but for example also by the rate of molecular transport towards the individual crystals and by the dissipation rate of the heat of sorption^3,4^.

Thus, it is certainly no coincidence that the so-called microscopic diffusion measurement techniques such as single-crystal/particle micro-imaging^11,12^ and quasi-elastic neutron scattering (QENS)^13,14^ are so far the only ones, which provided at least some evidence for the occurrence of such anomalies. Since here the measurements are made directly inside the crystals, they usually remain unaffected by interfering influences. However, given the great efforts generally involved in such measurements and the limits of their accuracy, we are still far away from a comprehensive picture of this phenomenon through experimental observation.

No question that, with this background, the experimental observation of Hyperloop-like diffusion of long-chain molecules under confinement^1^ is going to attract the particular interest of the community. A key result of this paper was that in a suitably sized 1-d-channeled zeolite (structure type TON) the diffusivity of n-dodecane (~8 × 10^–9^ m^2^s^–1^) was reported to significantly exceed the diffusivity (~1.2 × 10^–9^ m^2^s^–1^) of the much smaller n-butane molecules. Such an anomaly could indeed be easily attributed to the fact that the longer molecules – like in a hyperloop – can follow the centerline of the channels more easily than butane, whose shortness makes it much more prone to canting. With increasing pore diameter, this advantage may be expected to be lost, and this tendency is indeed reported to be observed in both measurements and molecular dynamic simulations for MTW and AFI type zeolites.

For experimentalists, as a matter of course, there remains the question of whether the dependencies observed in the experiment can indeed be attributed to diffusion and thus to the suspected anomalies in diffusion behavior. Using data provided by the authors the curves given in Fig. 5e^1^ were replotted as normal uptake curves. The dual resistance model curves are used as these were regressed on the original data and smooth out the signal noise. They are shown in Fig. 1 for the three zeolites, along with a pure exponential curve that allows to unequivocally determine the time constants of these experiments, yielding 87, 546, and 971 s for TON, MTW, and AFI, respectively. The derived square root of time plot clearly shows that it is not a diffusion process, while the exponential decay plot provides clear evidence of the first-order dynamic response of the system. This indicates that it is not possible to apply the dual resistance model to the experiments as only one resistance can be identified.

**Fig. 1:**
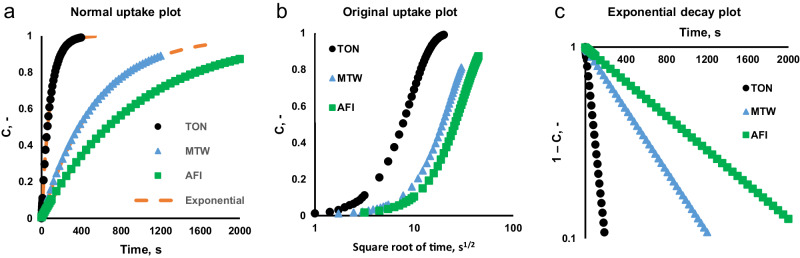
Different ways to plot uptake curves reveal single mechanism. Normal uptake plots (**a**), i.e. dimensionless concentration C vs time, for the three materials (TON – black circles, MTW – blue triangles, and AFI – green squares) replotted from ref. ^1^. along with exponential match for estimating the time constants of molecular uptake of n-dodecane. The derived square root of time plot (**b**) (original plot in ref. ^1^) does not provide a clear indication of the controlling mechanism, while the exponential decay plot (**c**) demonstrates that there is only one mechanism being observed and it is not a diffusion process. Source data are provided as a Source Data file.

The authors did not specify the dimension used to convert the diffusion time to the diffusivity in the original paper, but have provided the following values which are consistent with SEM images of the TON and AFI crystals included in Supplementary Fig. 13^1^. The crystal lengths used are *L*_TON_ = 1 μm; *L*_AFI_ = 2 μm, and *L*_MTW_ = 1 μm. From the values of intracrystalline diffusivities, D, reported in Fig. 5d^1^ it is therefore possible to estimate the time constants (first statistical moment) of molecular uptake under diffusion limitation as

1 $${\left(\frac{{L}^{2}}{12D}\right)}_{{{\mbox{TON}}}}=\;0.01\,{{{{{\rm{ms}}}}}}{\;\;\;}{\left(\frac{{L}^{2}}{12D}\right)}_{{{\mbox{MTW}}}}=\;0.02\,{{{{{\rm{ms}}}}}}{\;\;\;}{\left(\frac{{L}^{2}}{12D}\right)}_{{{\mbox{AFI}}}}=\;0.16\,{{{{{\rm{ms}}}}}}$$

The rate of equilibration by diffusion is thus seen to be at least four orders of magnitude faster than the overall uptake rate. The same conclusion applies to the butane data kindly provided by the authors. Therefore, the rate of molecular uptake is unaffected by intracrystalline diffusion, a conclusion already strongly suggested by the fact that the experimentally determined sorption curves are very well approximated by exponential decays - in contrast to what would normally be the case with diffusion limitation.

The comprehensive demonstration of the occurrence of the anomalies predicted in the molecular dynamics simulations undoubtedly remains a compelling task for experimentalists. The chances of success will be greater if we adhere to principles of good experimental practice in the future. This highlights the importance and timeliness of an IUPAC initiative, which has been dedicated to provide a first comprehensive set of guidelines for measurements and reporting of diffusion properties of chemical compounds in nanoporous materials serving for catalytic, mass separation, and other relevant purposes^15^.

### Source data

Source data file

## Data Availability

All data needed to evaluate the conclusions of this study are included in the Source Data file. Source data are provided with this paper.

## References

[1] Yuan J (2023). Hyperloop-like diffusion of long-chain molecules under confinement. Nat. Commun..

[2] Gorring RL (1973). Diffusion of normal paraffins in zeolite T occurrence of window effect. J. Catal..

[3] Ruthven DM (2006). The window effect in zeolitic diffusion. Micropor. Mesopor. Mater..

[4] Wang J-Y, Mangano E, Brandani S, Ruthven DM (2021). A review of common practices in gravimetric and volumetric adsorption kinetic experiments. Adsorption.

[5] Krishna R, van Baten JM (2009). A molecular simulation study of commensurate–incommensurate adsorption of n -alkanes in cobalt formate frameworks. Mol. Simul..

[6] Dubbeldam D, Calero S, Maesen TLM, Smit B (2003). Incommensurate diffusion in confined systems. Phys. Rev. Lett..

[7] Runnebaum RC, Maginn EJ (1997). Molecular dynamics simulations of alkanes in the zeolite silicalite - evidence for resonant diffusion effects. J. Phys. Chem. B.

[8] DEROUANE E (1988). Surface curvature effects in physisorption and catalysis by microporous solids and molecular sieves. J. Catal..

[9] Bhide SY, Yashonath S (2004). Anomalous diffusion of linear and branched pentanes within zeolite NaY. Mol. Phys..

[10] Yashonath S, Ghorai PK (2008). Diffusion in nanoporous phases: size dependence and levitation effect. J. Phys. Chem. B.

[11] Hwang S (2018). Anomaly in the chain length dependence of n-alkane diffusion in ZIF-4 metal-organic frameworks. Molecules.

[12] Kärger J (2014). Microimaging of transient guest profiles to monitor mass transfer in nanoporous materials. Nat. Mater..

[13] Borah BJ, Jobic H, Yashonath S (2010). Levitation effect in zeolites: quasielastic neutron scattering and molecular dynamics study of pentane isomers in zeolite NaY. J. Chem. Phys..

[14] Jobic H, Methivier A, Ehlers G, Farago B, Haeussler W (2004). Accelerated diffusion of long-chain alkanes between nanosized cavities. Angew. Chem. -Int. Ed..

[15] Kärger J, Ruthven DM, Valiullin R (2021). Diffusion research with nanoporous material: more than just a random walk?. Chem. Int..

